# Methods to include persons living with HIV not receiving HIV care in the Medical Monitoring Project

**DOI:** 10.1371/journal.pone.0219996

**Published:** 2019-08-01

**Authors:** Stanley C. Wei, Lauren Messina, Julia Hood, Alison Hughes, Thomas Jaenicke, Kendra Johnson, Leandro Mena, Susan Scheer, Chi-Chi Udeagu, Amy Wohl, McKaylee Robertson, Joseph Prejean, Mi Chen, Tian Tang, Jeanne Bertolli, Christopher H. Johnson, Jacek Skarbinski

**Affiliations:** 1 Division of HIV/AIDS Prevention, Centers for Disease Control and Prevention, Atlanta, Georgia, United States of America; 2 Oak Ridge Institute for Science and Education, Oak Ridge, Tennessee, United States of America; 3 Prevention Division, Public Health Seattle and King County, Seattle, Washington, United States of America; 4 HIV Epidemiology and Surveillance Section, San Francisco Department of Public Health, San Francisco, California, United States of America; 5 Office of Infectious Disease, Washington State Department of Health, Tumwater, Washington, United States of America; 6 STD/HIV Office, Mississippi State Department of Health, Jackson, Mississippi, United States of America; 7 Bureau of HIV/AIDS Prevention and Control, New York City Department of Health and Mental Hygiene, New York City, New York, United States of America; 8 Program Evaluation Unit, Los Angeles County Department of Public Health, Los Angeles, California, United States of America; University of Washington, UNITED STATES

## Abstract

The Medical Monitoring Project (MMP) is an HIV surveillance system that provides national estimates of HIV-related behaviors and clinical outcomes. When first implemented, MMP excluded persons living with HIV not receiving HIV care. This analysis will describe new case-surveillance-based methods to identify and recruit persons living with HIV who are out of care and at elevated risk for mortality and ongoing HIV transmission. Stratified random samples of all persons living with HIV were selected from the National HIV Surveillance System in five public health jurisdictions from 2012–2014. Sampled persons were located and contacted through seven different data sources and five methods of contact to collect interviews and medical record abstractions. Data were weighted for non-response and case reporting delay. The modified sampling methodology yielded 1159 interviews (adjusted response rate, 44.5%) and matching medical record abstractions for 1087 (93.8%). Of persons with both interview and medical record data, 264 (24.3%) would not have been included using prior MMP methods. Significant predictors were identified for successful contact (e.g., retention in care, adjusted Odds Ratio [aOR] 5.02; 95% Confidence Interval [CI] 1.98–12.73), interview (e.g. moving out of jurisdiction, aOR 0.24; 95% CI: 0.12–0.46) and case reporting delay (e.g. rural residence, aOR 3.18; 95% CI: 2.09–4.85). Case-surveillance-based sampling resulted in a comparable response rate to existing MMP methods while providing information on an important new population. These methods have since been adopted by the nationally representative MMP surveillance system, offering a model for public health program, research and surveillance endeavors seeking inclusion of all persons living with HIV.

## Introduction

The National HIV Surveillance System (NHSS) collects surveillance data on HIV infection in the United States. The Centers for Disease Control and Prevention’s (CDC’s) Medical Monitoring Project (MMP) provides complementary information about clinical outcomes of HIV infection; behaviors of persons living with HIV with respect to care seeking and care utilization, which affect prevention of HIV-related morbidity; and ongoing risk behaviors, which affect further transmission of HIV. MMP provides important national HIV prevention indicators such as the proportion of persons living with HIV in care who are prescribed antiretroviral therapy (ART).[1, 2]

When MMP was first proposed in 2004, HIV case reporting was not yet mandatory in all states; comprehensive rosters of persons diagnosed with HIV from which a sample could be drawn did not exist. Although early proponents of MMP preferred a system that sampled from among all HIV-diagnosed persons [3], it was not yet feasible to generate such a nationally representative sample using NHSS. However, it was possible to generate lists of HIV facilities and HIV patients being seen in each of those facilities, so a facility-based sampling method was employed for MMP [4]. In relying on HIV care providers for a list of potential participants, MMP necessarily excluded those who did not regularly visit HIV care providers. Those not regularly receiving HIV care are more likely to have detectable HIV virus in their bloodstream [5, 6], face higher mortality [7–9] and carry greater risk of transmitting HIV [10] than their in-care counterparts. Numerous studies have pointed out the limitations of existing data systems for guiding prevention among those not receiving HIV care [11, 12], and in its 2012 report assessing the adequacy of HIV-related data collection systems for providing data to guide HIV prevention, the Institute of Medicine specifically recommended that MMP broaden its target population to include persons not receiving HIV care.[13]

As of April 2008, all 50 states, the District of Columbia, and 6 dependent areas had fully implemented confidential name-based HIV surveillance and submit de-identified data on persons living with HIV to CDC. In this manuscript, we describe the Case-Surveillance-Based Sampling (CSBS) demonstration project, a robust multi-site pilot of an alternative sampling strategy for MMP in which persons were sampled from NHSS to determine the feasibility of using NHSS to include the population not receiving HIV care. We discuss the development of methods that make representative data collection on the population not receiving HIV care newly feasible. We present data on response rates for this pilot system, identify independent predictors of non-response, and describe methods of adjustment for non-response. Finally, we discuss the recently launched national implementation of CSBS sampling methods for MMP, and the implications of these methods for other public health program, research, and surveillance efforts.

## Materials and methods

### 2.1 Ethics Statement

CSBS, as a public health surveillance activity, was determined not to be research in accordance with the federal human subjects protection regulations at 45 Code of Federal Regulations 46.101c and 46.102d and CDC's Guidelines for Defining Public Health Research and Public Health Non-Research. Participating states or municipalities and facilities obtained local Institutional Review Board (IRB) approval to conduct CSBS when locally required.

### 2.2 Project area selection

All MMP project areas were eligible to apply for participation in the CSBS demonstration project[14]. Five project areas were selected by an objective external review panel; the selected areas were Los Angeles County (LAC), Mississippi (MS), New York City (NYC), San Francisco (SFO), and Washington State (WA). Diversity criteria were taken into account in an effort to make the results of the demonstration project relevant to all MMP jurisdictions and to ensure that challenges to successful recruitment would be included and evaluated. For example, one of the project areas did not have mandatory CD4+ T-lymphocyte cell count and viral load reporting at the time of application (MS); one of the areas required review by an Institutional Review Board (LAC); one of the areas had achieved below the mean MMP response rate in the most recent MMP data collection cycle (NYC); and two of the areas were geographically medium or large states (WA and MS). Funded project areas collected data concurrently for CSBS and MMP.

### 2.3 Eligibility & sampling for CSBS

To maximize ease of recruitment and relevancy of the public health data, we selected representative samples of all living adults with HIV according to their most recently reported address as reported through NHSS [15, 16]. Samples were drawn using SAS Versions 9.2 and 9.3 (SAS Institute, Cary, NC). Inclusion and exclusion criteria were assessed using only case surveillance data and with reference to a specific sampling date. This date was chosen based on the date of export of the case surveillance data used for sampling. We included potential participants who 1.) were aged 18 years and older, 2.) were presumed living, 3.) had been reported to CDC as a case of HIV[17] [18] and 4.) whose most recently reported residence fell within a CSBS project area. Residence was determined at the level of the public health jurisdiction; full address is not transmitted to CDC. Most recently reported residence was determined after consideration of all residence information reported through case surveillance including residence at HIV diagnosis, residence at AIDS diagnosis, and residence at the time of laboratory specimen collection or care visit, if available. The most recent available case surveillance data sets were used. The 2012 samples were drawn using *local* case surveillance data exported in September, 2012. At that time, the national NHSS analytic dataset retained only selected addresses rather than all reported addresses, and there was no calculated most recent address variable. For the 2013 and 2014 samples, we used *national* case surveillance data transmitted to CDC. De-duplication is a process by which potential duplicate cases in the national data are identified based on a matched combination of three variables including last name soundex (an alphanumeric code representing a person’s last name), date of birth, and sex at birth, but have not been confirmed as same or different by reporting jurisdictions. Case information is then sent to the jurisdictions from which they were reported, and after investigation the jurisdictions report back to CDC whether the reported cases represent the same, or different persons. These data had the advantage over the 2012 samples of combining data from all NHSS jurisdictions and having undergone national de-duplication. Persons who were recently diagnosed were oversampled. Those diagnosed in the past year, from 1–4 years ago, and five or more years ago made up 3%, 15% and 81% of the population, respectively. However, these subpopulations were sampled so as to make up 10%, 40%, and 50% of the sample, respectively.

We sampled between 200 and 300 persons per project area per year. The total sample size was 800 persons in 2012, 1000 persons in 2013, and 1010 persons in 2014 for a total of 2810 persons. Sample sizes were based on funding constraints; however, assuming a response rate of 33% and a design effect of 2, for estimates of proportion these sample sizes were expected to result in confidence interval half-widths of no greater than 0.051 for the total annual set of respondents and 0.090 for subgroup analyses involving 33% of the respondents.

### 2.4 Location and recruitment

Methods for locating sampled persons were tailored according to local data sources and regulations.[19] Participating health department staff linked sampled persons to additional health department and commercial databases using personal identifiers such as name and date of birth contained in local case surveillance records; personal identifiers were not transmitted to CDC for NHSS or CSBS.

**Fig 1:**
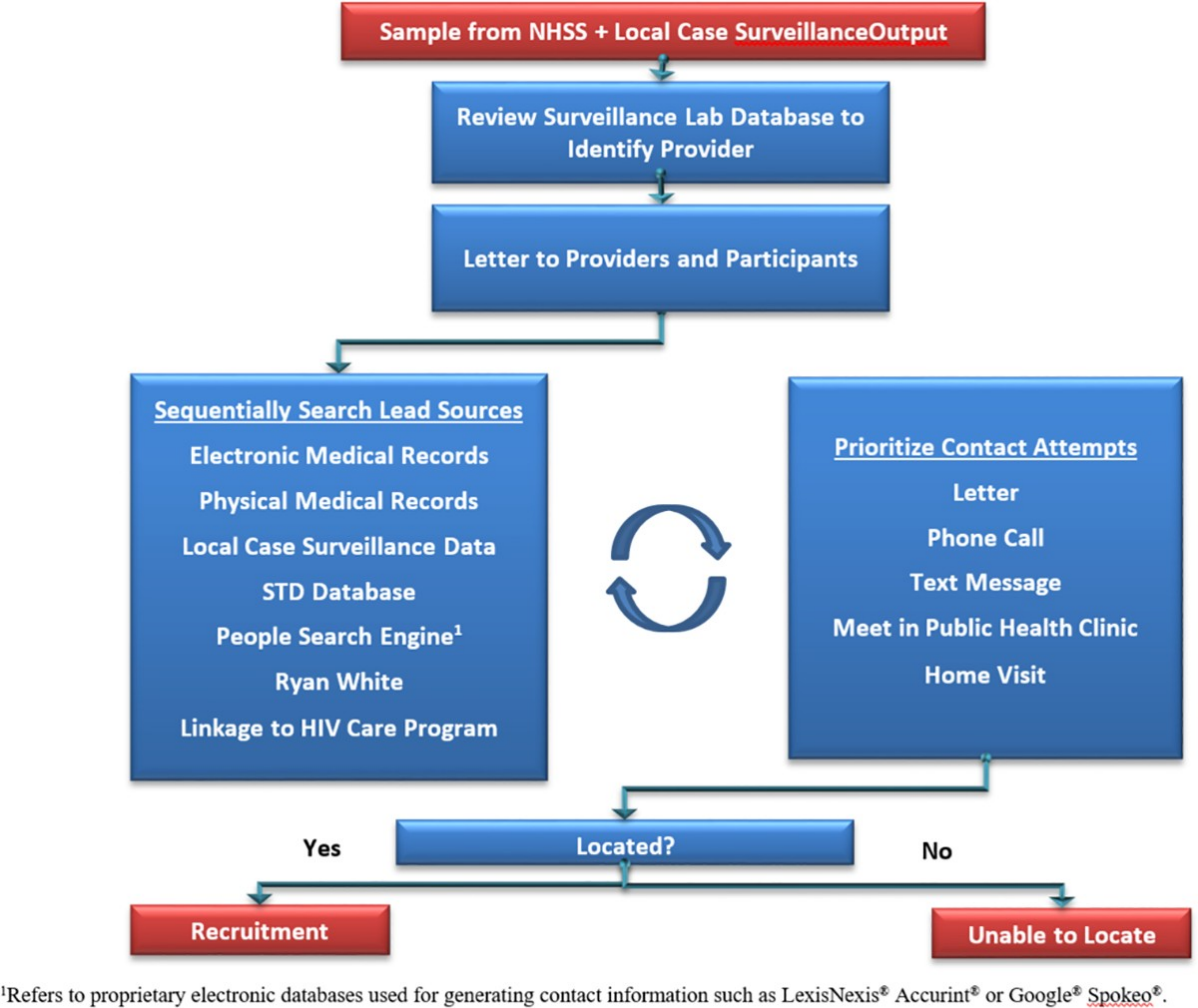
Example recruitment process for Case-Surveillance-Based Sampling demonstration project.

A flow diagram depicting typical recruitment procedures is included as Fig 1. CSBS project area staff generally began by attempting to identify a current HIV care provider through HIV-related laboratory reports to local case surveillance. If a provider was identified, then a letter was sent to the provider’s office informing him/her that a patient had been sampled for CSBS and providing an opportunity for the provider to voice any concerns about the specific patient being recruited to participate in CSBS. If no concerns, a letter of recruitment was sent by CSBS staff to the patient offering participation in a health survey without mention of HIV. Additional data sources were sequentially queried for addresses and phone numbers; the order in which data sources were used depended on the trade-off between accessibility of the data versus the expected benefit. HIV care providers were often asked to share current contact information as another potential data source if regulations allowed. As contact information was obtained, CSBS used standardized recruitment scripts to reach out to sampled persons using telephone calls, text messages, letters, and home visits, again in approximate order of decreasing utility and increasing resource intensity. Efforts were also made to meet the sampled person at upcoming public health clinic appointments prior to discontinuing attempts to locate the sampled person. In recognition of the fact that data sources are updated and availability via a given contact method may vary, staff repeatedly queried available data sources and re-contacted persons as additional contact information became available.

Interviewers verified eligibility of contacted persons according to age and HIV status, and noted sampled persons ineligible due to death as determined through lead sources (Fig 1). Persons who self-reported not residing in the project area on the date of recruitment were excluded in 2012 and considered ineligible. However, in the 2013 and 2014 data collection cycles, these persons were included as eligible after new procedures were implemented in consultation with the HIV subcommittee of the Council of State and Territorial Epidemiologists and all US HIV surveillance coordinators to remotely collect data on these persons—considered newly eligible—when agreed upon in writing by the jurisdiction of current residence.

### 2.5 Data collection

Data were collected via face-to-face or telephone interviews and medical record abstraction (MRA) from November 2012 to May 2013, August 2013 to June 2014, and July 2014 to April 2015 for the 2012, 2013, and 2014 data collection cycles, respectively. Data collection methods were similar to those previously described for MMP[14] except for the following changes. MRAs were performed at the outpatient HIV care facility that the participant reported visiting most often in the year prior to the last care visit. If the sampled person could not be interviewed, did not identify a care facility, or denied a history of receiving HIV care, available case surveillance and electronic medical records were reviewed for evidence of HIV care. If present, medical records were abstracted under the state’s public health surveillance authority from the facility in which the sampled person most often received care. Interview and MRA data were collected with reference to a twelve-month surveillance period preceding the interview date if interviewed or the date of first contact attempt if not interviewed. Selected data elements were exported on all sampled persons from the national HIV case surveillance dataset compiled from data transmitted to CDC one year after the sampling date.

### 2.6 Security and confidentiality procedures

CSBS data were subject to the same security and confidentiality requirements as used for HIV case surveillance data at state and local project areas, as well as at CDC. These requirements included adherence to CDC’s Data Security and Confidentiality Guidelines for HIV, Viral Hepatitis, Sexually Transmitted Disease, and Tuberculosis Programs [20]. CSBS interview and MRA data records transmitted to CDC did not contain specific participant identifiers (e.g., name, address, social security number) and were linkable to case surveillance data only through the case surveillance coded identifier. When attempts were made to contact sampled persons, the identities of responding persons were verified prior to discussion of HIV by confirmation of at least two pieces of personal information obtained from case surveillance, e.g., date of birth and former address.

### 2.7 Analytical cohort

The analytical cohort used for CSBS analyses and weighting was defined as those persons who provided complete demographic interview data, whether or not the interview was complete. MRA data, when present, and associated NHSS data were contained in linked relational tables. Data quality problems resulted in the exclusion of all Mississippi data. All descriptive and analytic statistics were computed using SAS, Version 9.3.

### 2.8 Recruitment outcome definitions

CSBS recruiters systematically tracked data sources queried and recruitment outcomes in a dedicated database. Sampled persons were considered to have been located if any of three situations applied: 1.) the sampled person was successfully contacted by the interviewer; 2.) a highly reliable data source, i.e., either a statement from a knowledgeable third party such as a relative or health department worker, or documentation in medical, correctional, or vital records, indicated the person resided outside of the project area at the time of recruitment in a jurisdiction where data collection was not permitted; 3.) two or more reliable data sources, e.g., HIV laboratory surveillance reports and people search engines such as Accurint (LexisNexis, Dayton, OH) and Spokeo (Google, Mountain View, CA), indicated the same residence in the same location outside of the project area at the time of recruitment in a jurisdiction where data collection was not permitted.

Adjusted participant response rates were calculated using American Association for Public Opinion Research formula RR3 accounting for participants of unknown eligibility as follows:

$$\mathrm{Response}\;\mathrm{Rate}=\frac{\#Respondents}{\#Respondents+eligible\;nonrespondents+(e)(persons\;of\;unknown\;eligibility\;status)}$$ [1]

where e was the proportion eligible among those of known eligibility status [21]. Facility-based MMP response rates, included for comparison to CSBS, were computed as the product of the adjusted participant response rate as defined above and the facility response rate. Recruiters systematically recorded reactions of sampled persons to recruitment attempts on a five-point scale from strongly negative to strongly positive (2013) as well as adverse events, defined as events reported to or observed by any study staff leading to substantial psychological, social, or physical harm to a participant that resulted from his or her participation in the study. Participants were compensated approximately $40 in cash or the equivalent for participation; compensation amounts differed slightly between project areas but were consistent between CSBS and traditional MMP projects conducted in the same project area.

### 2.9 HIV care history definitions

For analytical purposes, persons who self-reported never having received HIV care and who had no MRA evidence of HIV care were classified as never having received HIV care. Interviewed persons were considered to be receiving sufficient care to be included in facility-based MMP if they made at least one visit to an HIV care provider in the first four months of the surveillance period as recorded by MRA [4], regardless of self-reported care history[14, 22]. The MMP 4-month population definition period is based on analyses showing that 88% of patients seen in 2003 were seen within the first 4 months of the year [23]. HIV care received by sampled persons was also classified according to the Health Resources and Services Administration (HRSA) performance measure for medical visits, i.e., those whose medical records showed at least two visits with an HIV care provider in the last 12 months that were at least 90 days apart were considered to be in continuous care [24]. We defined an HIV care provider as a person who was licensed to prescribe HIV antiretroviral therapy. The HIV care history for persons who self-reported having received HIV care but had no associated MRA was classified as unknown. All interviewed persons who self-reported having not seen an HIV outpatient care provider in the past six months were offered assistance with engagement in HIV care, typically by referral to existing HIV public health programs.

### 2.10 Methods for evaluation and minimization of bias

We calculated analytical weights to account for five primary issues: 1.) unequal selection probabilities resulting from stratified random sampling, 2.) over-inclusion of persons on the sampling frame due to incorrect data, e.g., incorrect vital status, HIV status, or age on the sampling date 3.) non-response bias, 4.) under-inclusion of persons on the sampling frame due to HIV case reporting delay, and 5.) inflation of variance estimates due to extreme weights. We weighted data from each project area and year separately due to the possibility that factors predicting non-response could vary. To illustrate typical associations, we describe predictors of non-response and case-reporting delay using models based on 2014 data for Washington State as an example (Table 1). Tables describing the variables included in final models for other project areas and other data years are presented as supplementary tables (S1, S2 and S3 Tables). Predictors were categorical, ordinal, or continuous. For categorical variables with multiple levels such as race, we used dichotomous indicator variables, which allowed multi-level categories to be reduced to fewer categories during model selection.

**Table 1:** Bivariate and multivariate associations between potential predictors and two recruitment outcomes, successful contact and successful interview among contacted persons, using data from Washington State, Case-Surveillance-Based Sampling demonstration project, 2014.

**Categorical or Ordinal Predictor**	**Levels**	**Contacted, n (%)**	**Not Contacted, n (%)**	**Bivariate Model, OR (95% CI)**^**1**^	**p-value**^**2**^	**C Statistic**	**Multivariate Model, adjusted OR (95% CI)**	**Interviewed, n (%)**	**Not Interviewed, n (%)**	**Bivariate Model, OR (95% CI)**^**1**^	**p-value**^**2**^	**C Statistic**	**Multivariate Model, adjusted OR (95% CI)**
AIDS Status	Not AIDS	92 (62.6)	55 (37.4)	0.53 (0.25–1.12)	**0**.**05**	0.58	0.58 (0.23–1.48)	65 (70.7)	27 (29.4)	1.42 (0.63–3.23)	0.62	0.53	Not included
	Immunologic	74 (75.5)	24 (24.5)	0.97 (0.43–2.20)			0.71 (0.24–2.06)	53 (71.6)	21 (28.4)	1.49 (0.64–3.50)			
	Clinical	35 (76.1)	11 (23.9)	REF			REF	22 (62.9)	13 (37.1)	REF			
Care Status	In Care (HRSA)^3^	144 (81.4)	33 (18.6)	**6.43 (3.21–12.88)**	**< .01**	0.69	**5.02 (1.98–12.73)**	100 (69.4)	44 (30.6)	1.33 (0.49–3.59)	0.71	0.53	Not included
	≥1 lab test in last 12 months but not in care	38 (56.7)	29 (43.3)	1.93 (0.91–4.12)			1.52 (0.62–3.70)	28 (73.7)	10 (26.3)	1.63 (0.50–5.31)			
	0 lab tests	19 (40.4)	28 (59.6)	REF			REF	12 (63.2)	7 (36.8)	REF			
Number jurisdictions reporting	1	159 (71.3)	64 (28.7)	1.54 (0.87–2.72)	0.14	0.54	1.17 (0.56–2.47)	107 (67.3)	52 (32.7)	0.56 (0.25–1.26)	0.16	0.54	Not included
	≥2	42 (61.8)	26 (38.2)	REF				33 (78.6)	9 (21.4)	REF			
Race/Ethnicity: Hispanic	No	176 (71.8%)	69 (28.2%)	REF	**0.02**	0.55	REF	120 (68.2%)	56 (31.8%)	REF	0.23	0.53	Not included
	Yes	25 (54.4%)	21 (45.7%)	**0.47 (0.03–0.15)**			0.63 (0.25–1.61)	20 (80.0%)	5 (20.0%)	1.85 (0.67–5.26)			
Race/Ethnicity: Non-Hispanic Black	No	169 (68.7%)	77 (31.3%)	REF	0.75	0.51	Not included	116 (68.6%)	53 (31.4%)	REF	0.47	0.52	REF
	Yes	32 (71.1%)	13 (28.9%)	1.12 (0.56–2.27)				24 (75.0%)	8 (25.0%)	1.37 (0.58–3.23)			1.29 (0.52–3.23)
Race/Ethnicity: Non-Hispanic White	No	74 (61.7%)	46 (38.3%)	REF	**0.02**	0.57	REF	52 (70.3%)	22 (29.7%)	REF	0.88	0.51	Not included
	Yes	127 (74.3%)	44 (25.7%)	**1.79 (1.09–2.94)**			1.41 (0.68–2.92)	88 (69.3%)	39 (30.7%)	0.95 (0.51–1.79)			
Residence in project area of sampling	No or Unknown	83 (50.3)	82 (49.7)	**0.07 (0.03–0.15)**	**< .01**	0.75	**0.08 (0.04–0.19)**	44 (53.0)	39 (47.0)	**0.26 (0.14–0.49)**	**< .01**	0.66	**0.24 (0.12–0.46)**
	Yes	118 (93.7)	8 (6.4)	REF			REF	96 (81.4)	22 (18.6)	REF			REF
Sex	Male	172 (68.5)	79 (31.5)	REF	0.61	0.51	REF	120 (69.8)	52 (30.2)	REF	0.93	0.50	Not included
	Female	29 (72.5)	11 (27.5)	1.21 (0.58–2.55)			1.23 (0.51–2.94)	20 (69.0)	9 (31.0)	0.96 (0.41–2.26)			
Transmission risk	Not MSM^4^	61 (63.5)	35 (36.5)	0.68 (0.41–1.15)	0.15	0.54	Not included	40 (65.6)	21 (34.4)	0.76 (0.40–1.45)	0.41	0.53	Not included
	MSM	140 (71.8)	55 (28.2)	REF				100 (71.4)	40 (28.6)	REF			
**Continuous Predictor**	**Units**	**Contacted, mean (SD)**	**Not Contacted, mean (SD)**	**Bivariate Model, OR (95% CI)**^**1**^	**p-value**^**5**^	**C Statistic**	**Multivariate Model, adjusted OR (95% CI)**	**Interviewed, mean (SD)**	**Not Interviewed, mean (SD)**	**Bivariate Model, OR (95% CI)**^**1**^	**p-value**^**5**^	**C Statistic**	**Multivariate Model, adjusted OR (95% CI)**
Age at HIV diagnosis	Years	36.6 (10.7)	35.8 (10.8)	1.01 (0.98–1.03)	0.57	0.53	1.03 (0.95–1.13)	35.9 (10.2)	38.2 (11.7)	0.98 (0.95–1.01)	0.1499	0.552	Not included
Age on sampling date (Years)	Years	45.3 (11.6)	43.6 (12.1)	1.01 (0.99–1.03)	0.26	0.55	0.96 (0.88–1.05)	44.8 (11.7)	46.4 (11.3)	0.99 (0.96–1.01)	0.3504	0.547	0.97 (0.94–1.00)
Contact information age (Years)	Years	6.7 (5.5)	5.8 (4.9)	1.03 (0.98–1.09)	0.17	0.54	1.07 (0.94–1.21)	6.8 (5.5)	6.4 (5.4)	1.01 (0.96–1.07)	0.6309	0.526	Not included
Time since diagnosis	Years	8.7 (7.5)	7.8 (6.8)	1.02 (0.98–1.05)	0.31	0.54	Not included	8.9 (7.7)	8.2 (7.3)	1.01 (0.97–1.05)	0.5447	0.524	1.03 (0.99–1.08)

^1^ Confidence intervals that exclude the null value, indicating statistical significance, are shown in bold.

^2^ P-value for chi-squared test of general association, values ≤0.05 are shown in bold.

^3^ Modified Health Resources and Service Administration definition, ≥ 2 HIV-related laboratory tests ≥ 90 days apart in the 12 months prior to the sampling date

^4^ Men who have sex with men

^5^ P-value for test of hypothesis that row means scores do not differ, values ≤0.05 are shown in bold.

We first calculated design weights for all sampled persons within time-since diagnosis strata as the inverse of the probability of selection. Second, we removed from the analytical dataset persons deemed ineligible in the course of attempted recruitment. By removing ineligible persons from the dataset following design weighting, we in effect corrected the sampling frame for ineligible persons who were mistakenly placed on the sampling frame. To maintain consistency in the population of inference, residency out of jurisdiction did not make persons ineligible for weighting purposes in any year, i.e., all out-of-jurisdiction sampled residents in 2012 were eligible non-responders for weighting purposes.

The first form of non-response that we considered was failure to contact the sampled person. We considered 13 potential predictors of successful contact as described in Table 1. With the exception of residence outside the jurisdiction of sampling, which was collected by the recruiter, all predictor data were obtained from NHSS. We examined the associations of predictors with non-response using chi-square tests,odds ratios, and area under the curve (AUC or c-statistic); AUC was calculated using logistic regression for the bivariate relationship between predictors and an indicator variable for successful contact. As an initial screen, we retained only those predictors whose association with contact was significant at p<0.10 for at least one project area. Next we defined a multivariable logistic regression model by serially eliminating predictors from the model with the lowest AUC until the model converged and parameter estimates were stable. We used this final model to break the sample into five quintiles of propensity for contact, and we adjusted the design weight among contacted persons according to the actual proportion contacted within each quintile.

We then adjusted for a second form of non-response, failure to obtain an interview from successfully contacted persons. We used the same model selection procedure, except using completed interview as the outcome rather than contact. We adjusted the weights calculated above among contacted respondents according to the actual proportion responding within each quintile of propensity for interview.

Our adjustment for case reporting delay rested on the observation that current NHSS reporting delays rarely exceed one year. We used case surveillance data transmitted one year after the sampling date to construct a corrected frame using the same inclusion criteria as for the initial frame. To be consistent with sampling methods, in 2012 local case surveillance data were used, and in 2013 and 2014 national data were used. We used this new frame to post-stratify the data, updating the original sampling frame. These strata were defined in a manner analogous to that used for non-response adjustment, except we chose different candidate predictor variables (Example from Washington State shown in Table 2) such as date of diagnosis and urbanicity based on the variables used in calculation of NHSS reporting delay weights [25]. To be precise, this form of adjustment for case reporting delay also adjusts for other types of updates to the NHSS system affecting eligibility. The list of eligible people constructed from more mature data may differ from the original list due to both additions and subtractions as a different set of persons are considered to have met all four eligibility criteria as of the sampling date.

**Table 2:** Bivariate and multivariate associations between potential predictors and HIV case reporting delay using pooled data from Washington State, Case-Surveillance-Based Sampling demonstration project 2014.

**Categorical Predictor**	**Levels**	**Delayed, n (%)**	**Not Delayed, n (%)**	**Bivariate Model, OR (95% CI)**^**2**^	**p-value**^**3**^	**C Statistic**	**Multivariate Model, aOR (95% CI)**
AIDS History: AIDS Diagnosed After HIV	No	86 (1.2%)	7208 (98.8%)	REF	**< .01**	0.61	REF
	Yes	15 (0.3%)	4341 (99.7%)	**0.29 (0.17–0.50)**			0.55 (0.30–1.01)
AIDS History: AIDS Diagnosed Concurrent with HIV	No	87 (0.9%)	9258 (99.1%)	REF	0.13	0.53	REF
	Yes	14 (0.6%)	2291 (99.4%)	0.65 (0.37–1.15)			**0.52 (0.28–0.97)**
Facility of HIV Diagnosis: Inpatient	No	95 (0.9%)	10667 (99.1%)	REF	0.52	0.51	REF
	Yes	6 (0.7%)	882 (99.3%)	0.76 (0.33–1.75)			0.82 (0.34–2.00)
Facility of HIV Diagnosis: Other	No	70 (0.7%)	9693 (99.3%)	REF	0.57	<0.0001	REF
	Yes	31 (1.6%)	1856 (98.4%)	**2.33 (1.52–3.57)**			1.37 (0.87–2.17)
Facility of HIV Diagnosis: Missing	No	95(1.0%)	9699 (99.0%)	REF	**0.01**	0.55	REF
	Yes	6 (0.3%)	1850 (99.7%)	**0.33 (0.14–0.76)**			2.40 (0.96–6.02)
Race/Ethnicity: Hispanic	No	88 (0.9%)	10035 (99.1%)	REF	0.94	N/A^4^	Not included
	Yes	13 (0.9%)	1514 (99.2%)	0.98 (0.55–1.75)			
Race/Ethnicity: Non-Hispanic Black	No	83 (0.8%)	9781 (99.2%)	REF	0.49	0.51	REF
	Yes	18 (1.0%)	1768 (99.0%)	1.20 (0.72–2.00)			1.30 (0.76–2.24)
Race/Ethnicity: Non-Hispanic White	No	35 (0.8%)	4133 (99.2%)	REF	0.81	N/A^4^	Not included
	Yes	66 (0.9%)	7416 (99.1%)	1.05 (0.69–1.59)			
Race/Ethnicity: Other Non-White, Non-Black, Non-Hispanic Race	No	97 (0.8%)	10698 (99.2%)	REF	0.81	N/A^4^	REF
	Yes	4 (0.5%)	851 (99.5%)	0.52 (0.19–1.41)	0.19	0.52	0.45 (0.16–1.24)
Sex	Male	93 (0.9)	9918 (991)	1.91 (0.93–3.94)	0.07	0.53	1.30 (0.76–2.24)
	Female	8 (0.5)	1631 (99.5)	REF			REF
Transmission risk	MSM^5^	70 (0.9)	8161 (99.2)	**2.02 (1.01–4.06)**	**0.04**	0.55	1.53 (0.72–3.23)
	Other Identified Risk	9 (0.4)	2123 (99.6)	REF			REF
Urban Setting of HIV Diagnosis	Population < 2.5 Million	64 (1.4)	4436 (98.6)	**2.77 (1.85–4.16)**	**< .01**	0.63	**3.18 (2.09–4.85)**
	Population ≥ 2.5 Million	37 (0.5)	7113 (99.5)	REF			REF
**Continuous Predictor**	**Units**	**Delayed, mean (SD)**	**Not Delayed, mean (SD)**	**Bivariate Model, OR (95% CI)**^**2**^	**p-value**^**6**^	**C Statistic**	**Multivariate Model, aOR (95% CI)**
Age at HIV diagnosis	Years	39.6 (11.3)	34.7 (10.0)	**1.05 (1.03–1.06)**	**< .01**	0.63	**1.03 (1.01–1.05)**
HIV Diagnosis Date	Years^7^	50.0 (6.7)	41.4 (7.4)	**1.33 (1.26–1.41)**	**< .01**	0.86	**1.00 (1.00–1.00)**

^1^ Cases with delayed reporting were those present on a list of HIV cases generated using a newer version of the case surveillance dataset, but not on a list based on a version one year older.

^2^ Confidence intervals that exclude the null value, indicating statistical significance, are shown in bold.

^3^ P-value for chi-squared test of general association, values ≤0.05 are shown in bold.

^4^ Selection probabilities for the different levels of this variable are too similar so a c-statistic is not calculated, i.e., variable has very poor discrimination.

^5^ Men who have sex with men

^6^ P-value for test of hypothesis that row means scores do not differ, values ≤0.05 are shown in bold.

^7^HIV diagnosis date is expressed as years since January 1, 1960. A mean date of 50.0 years corresponds to December 31, 2009, and a mean date of 41.4 corresponds to May 26, 2001.

In order to prevent undue variance inflation resulting from the weighting procedures, we capped weights at a value equal to the median plus three times the interquartile range.[26, 27] Then we re-scaled weights so that totals would be consistent with the corrected sampling frame.

## Results

### 3.1 Recruitment outcomes

**Fig 2:**
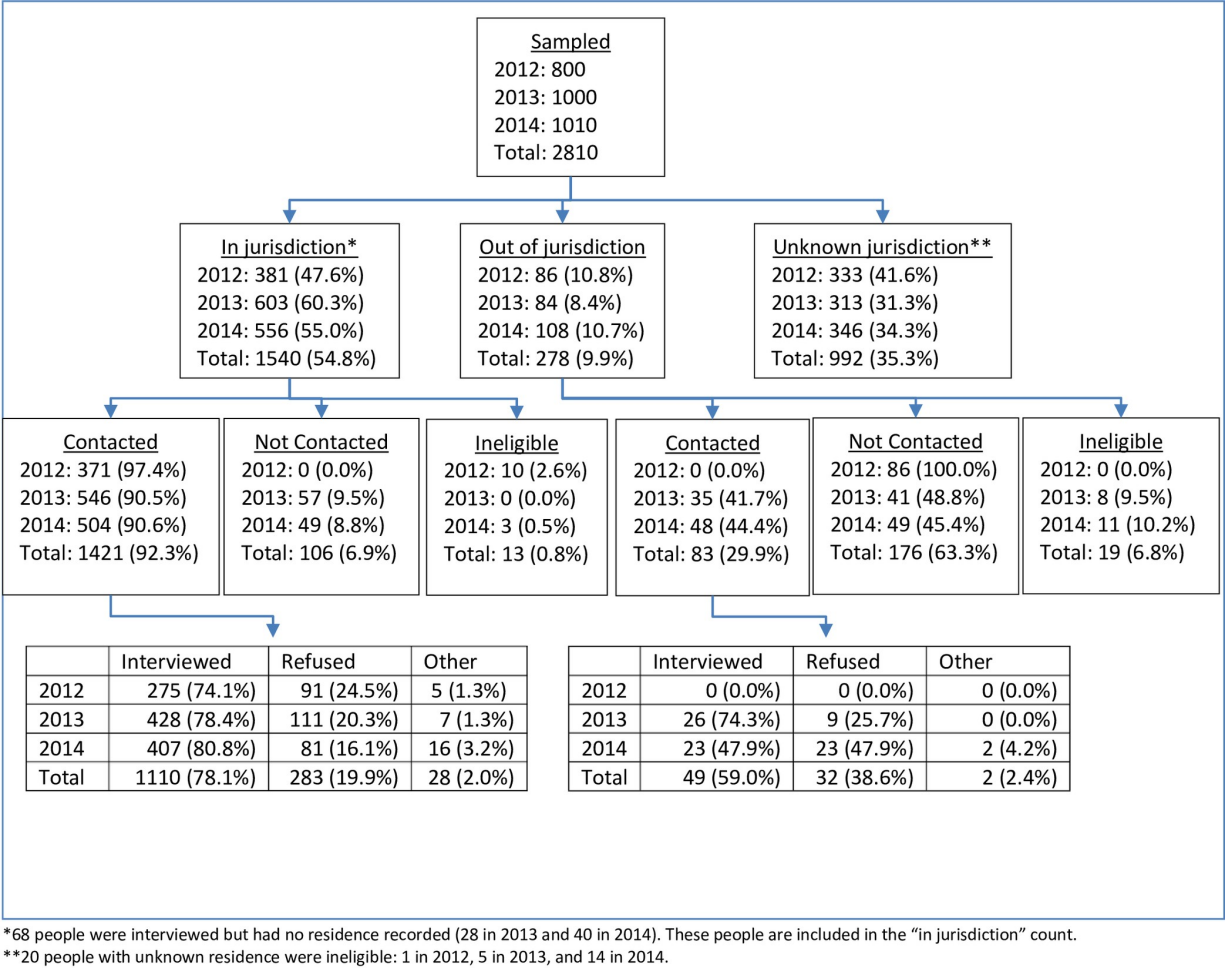
Search and interview recruitment outcomes for sampled persons, Case-Surveillance-Based Sampling demonstration project, 2012–2014.

Of 2810 persons sampled in 4 project areas in the 2012, 2013, and 2014 data collection cycles, 1812 (64.5%) were successfully located either in jurisdiction or out of jurisdiction (Fig 2). Of those, 1534 (84.7%) were known to reside in the project area in which they were sampled at the time of recruitment. Of these located in jurisdiction, 1421 eligible persons (92.3%) were contacted while 13 were ineligible—3 (0.2%), due to not being infected with HIV, and 10 (0.7%), due to residing outside of the country on the sampling date. Of contacted persons, 1110 (78.1%) were interviewed, and 283 (19.9%) refused. All persons residing out of jurisdiction were ineligible in 2012. In 2013 and 2014, 49 persons living in 13 states out of the jurisdiction of sampling were interviewed under newly developed procedures, making up 5.5% of persons interviewed in those years and contributing to the total of 1159 persons interviewed in all three years. Of 631 sampled persons in 2013 for whom recruiter assessments are available, 72 (11.4%) had a positive or strongly positive reaction to being recruited for CSBS, 518 (82.1%) had a neutral reaction to recruitment, and 41 (6.5%) had a negative or strongly negative reaction to recruitment; these data were not collected in 2014. Two contacted persons with a verified positive HIV diagnosis reported being unaware of that diagnosis and were linked to HIV care. No adverse events were reported.

**Fig 3:**
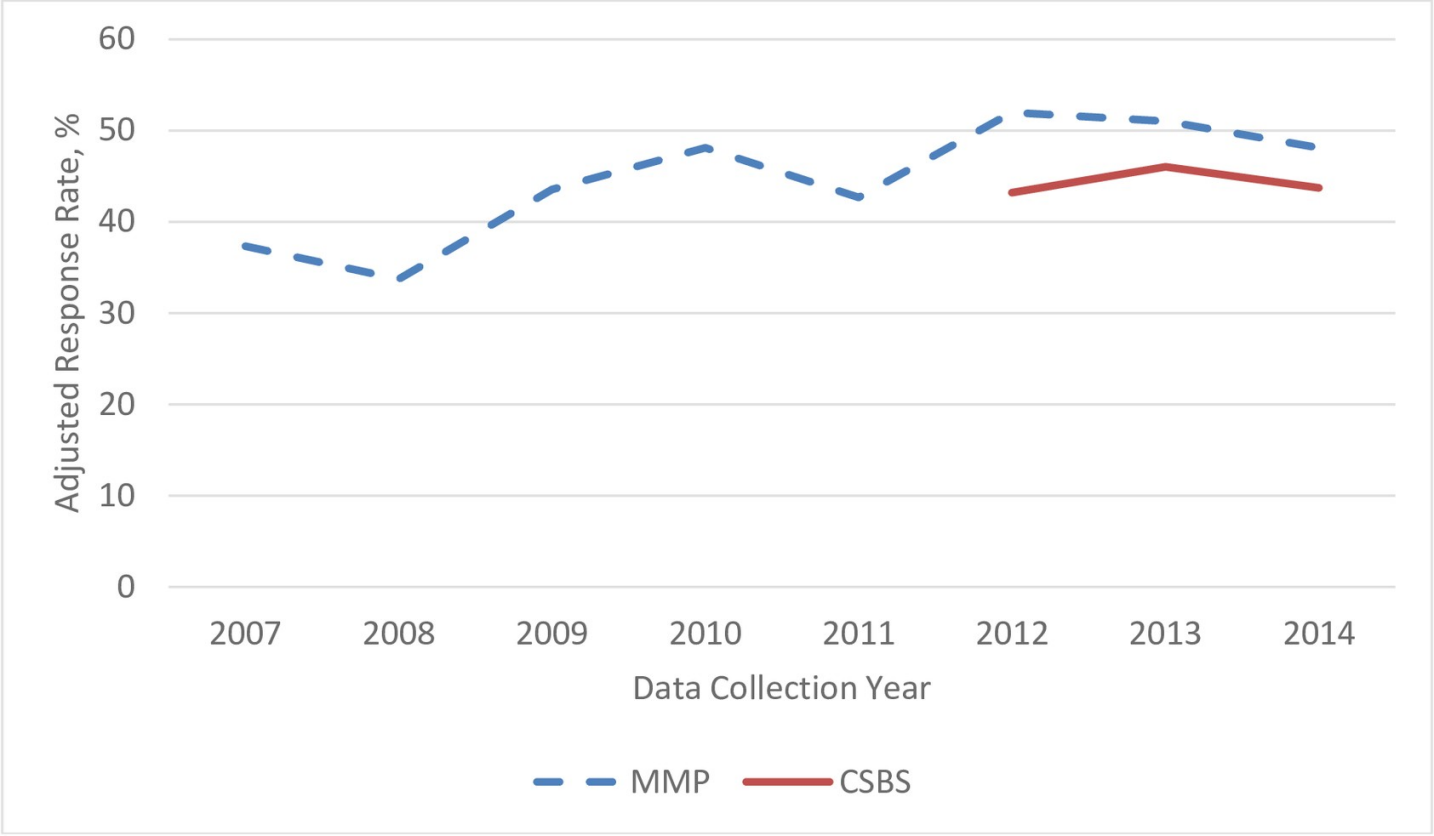
Comparison of adjusted interview response rates by data collection year in the facility-based Medical Monitoring Project (MMP) and the Case-Surveillance-Based Sampling (CSBS) demonstration project.

The adjusted interview response rate among eligible persons was 43.3%, 46.0%, and 43.7% in 2012, 2013, and 2014 respectively. Compared to response rates in MMP using facility-based sampling, response rates for CSBS were 8.7%, 5.0%, and 4.4% lower in 2012, 2013, and 2014, respectively (Fig 3). When CSBS data from the three years were combined, the adjusted interview response rate was 44.5%. Among all 1159 interviewed persons, 514 (44.4%) were interviewed by telephone, 216 (18.6%) were interviewed in health department offices, 156 (13.5%) were interviewed in an outpatient health facility, and 183 (15.8%) were interviewed in private homes.

In the combined years, the adjusted interview response rates by project area were 41.1% in Los Angeles, 41.8% in New York City, 42.6% in San Francisco, and 50.4% in Washington State. Of 1159 interviewees, 1146 persons (98.9%) self-reported a history of receiving HIV care, and project areas completed a matching MRA for 1087 (93.8%).

### 3.2 HIV care history

In the combined years, 80 out of 1133 (7.1%) interview respondents who provided information on HIV outpatient care self-reported not receiving care in the six months prior to interview and were offered linkage to care or re-engagement services through the CSBS project. Based on MRA, 54 participants (5.0%) had had no clinical visits in the past 12 months of whom 2 reported having never been in care at all (0.2%). In unweighted analysis, 264 (24.3%) CSBS respondents received insufficient care to be included in facility-based MMP surveillance (Table 3). By the HRSA performance measure, 202 (18.6% percent of all respondents) were not retained in care.

**Table 3:** HIV care as documented in the medical record for Case-Surveillance-Based Sampling respondents, 2012, 2013, and 2014 (N = 1087^1^).

		Retention in HIV Care Definition	
		MMP^2^ (%)	HRSA^3^ (%)
Retained in HIV care		823 (75.7)	885 (81.4)
Not retained in HIV care		264 (24.3)	202 (18.6)
	Some care (≥ 1 visit in past 12 months)	208 (78.9)	146 (72.3)
	No care in past 12 months	54 (20.5)	54 (26.7)
	Never in Care	2 (0.8)	2 (1.0)

^1^72 participants who reported a history of receiving HIV care but did not have medical record abstraction data to allow further classification are excluded.

^2^Medical Monitoring Project definition: Medical record showed at least one visit in the first four months of the 12-month medical record abstraction period.

^3^Health Resources and Services Administration definition: Medical record showed at least two visits in the 12-month medical record abstraction period at least 90 days apart.

### 3.3 Evaluation and minimization of bias

Recruitment success depended on many characteristics of the sampled persons; 2014 data from Washington State are presented for example in Table 1. Weighting-related data are not combined across project areas and years, because each project area was weighted independently each year. In 2014 Washington State, being in care was independently associated with success in contacting the sampled person (adjusted Odds Ratio [aOR] 5.02; 95% Confidence Interval [CI]: 1.98–12.73). Those known to reside outside the project area of sampling were also substantially less likely to be contacted than those residing inside the project area of sampling (aOR 0.08; 95% CI: 0.04–0.19). In bivariate analyses, Hispanic sampled persons were significantly less likely to be contacted than non-Hispanic sampled persons (Odds Ratio [OR] 0.47: 95% CI: 0.03–0.15) and non-Hispanic white sampled persons were more likely to be contacted than other race/ethnicity groups (OR 1.79; 95% CI: 1.09–2.94), but these effects were not statistically significant in multivariate analysis. Sex, age at HIV diagnosis, age on the sampling date, and transmission risk category were not significantly associated with contact. The final predictive model for contact in 2014 contained 10 of 13 candidate predictors (Table 1) and had an overall AUC of 0.85. Which variables were significantly associated with contact and were ultimately included in the model used for weighting varied by project area and data collection year (S1 Table).

Once the sampled person had been successfully contacted, the only variable that significantly predicted a successful interview in Washington State 2014 was place of residence (Table 1). Persons residing outside the project area of sampling or with unknown residence were less likely to be interviewed than those residing within the project area of sampling (aOR 0.24; 95% CI: 0.12–0.46). The final predictive model of successful interview for Washington State 2014 contained 4 predictors and had an AUC of 0.71. Again, which variables were significant and the composition of final models varied by project area and data collection year (S2 Table).

By comparing the sampling frame with a similarly constructed frame based on data from one year after the sampling date using data from 2014 in Washington State, we were able to identify persons who should have been on the initial frame but were not due to HIV case reporting delay. Sampled persons whose HIV case reports were delayed tended to be older than persons whose reports were not delayed with mean ages of 39.6 and 34.7, respectively (Table 2). Persons diagnosed with HIV in jurisdictions with populations less than 2.5 million were more likely to have delayed reporting than persons diagnosed in more populous jurisdictions (OR 2.77; 95% CI: 1.85–4.16). Men who have sex with men were more likely to have reporting delay than those with other reported risk (OR 2.02; 95% CI: 1.01–4.06). All 13 potential predictors were included in the final case reporting delay model, resulting in a model with AUC of 0.87. Variables included in the models for other project area/data collection year combinations are shown in S3 Table.

Our adjustment for case reporting delay and other changes to NHSS variables affecting eligibility is a form of post-stratification in which we update the sampling frame with a more accurate list of people living with HIV in the CSBS project areas. Cases of delayed reporting are added and cases whose updated information shows them to be ineligible are subtracted, e.g., those residing outside of the jurisdiction on the sampling date. For example, the sampling frame based on data transmitted to CDC from Washington State as of September 2014 contained 11,692 persons. A similarly constructed dataset based on data transmitted to CDC as of September 2015 contained 11,533 persons, an adjustment of -1.4%. Among the four project areas, the magnitude of the adjustment in 2014 ranged from -1.4% to 1.4%, averaging -0.1%.

## Discussion

While MMP has made significant contributions to the field of HIV surveillance,[28] its facility-based sampling method has limited its ability to support efforts to engage and re-engage out-of-care persons. CSBS offered proof of concept that MMP’s sampling methodology could be adapted to reach the broader population. Operating in multiple jurisdictions, CSBS established a nationally scalable solution for interview and medical record abstraction on a representative sample of all HIV-diagnosed persons both in and out of care with a response rate comparable to traditional MMP. In addition, we developed methods that directly address interjurisdictional migration, and differential non-response and reporting delays, reducing these potential sources of bias and allowing representative estimates to be made. These methods were particularly important in light of the literature showing bias when migration is not taken into consideration [29] or when clinic-, rather than population-based samples of out-of-care HIV-diagnosed persons are used [11].

In its third project year, CSBS achieved a response rate of 44.5%, within 4.4 percentage points of the overall MMP response rate, making it a viable alternative to facility-based sampling employed at that time for MMP, though the response rate is lower than optimal in both cases. A substantial proportion of respondents—approximately one quarter—would not have been included in MMP due to lower engagement in HIV care and 5% had no care in the past 12 months. These groups of respondents are likely to have lower levels of viral suppression, and understanding them may be particularly relevant to reducing HIV transmission and ultimately controlling the HIV epidemic in the United States. Based on these findings and the strategic importance of the population not receiving HIV care for HIV prevention, in June 2015, MMP incorporated the case-surveillance sampling-based methods described in this manuscript into its routine nationally representative data collection conducted in 17 states with a total sample size of 9700 for the 2015 cycle.

In the 2015 and 2016 cycles, following national adoption of CSBS methodology, MMP achieved response rates of 40% and 44%, respectively, very similar to the results of the pilot [30, 31]. In the twelve months prior to interview, 20.0% and 19.9% of respondents from each cycle, respectively, were not retained in care, and in the 24 months prior to interview, 36.0% were not retained in care in both cycles; retention was defined as having two or more elements of outpatient HIV care at least 90 days apart in each 12 month period. These further experiences demonstrate the feasibility of CSBS methods for collecting information from people living with HIV with irregular care patterns when scaled to a nationally representative sample of 23 state and local public health jurisdictions. While CSBS demonstrates that it is possible to identify and contact persons not retained in care through NHSS, the number of sampled persons not contacted was substantial, 1274 out of 2810 (45%), a reminder of the challenges facing Data to Care programs that seek to leverage NHSS to guide unretained patients back into care.

Sampling for MMP from NHSS and post stratification of MMP data to NHSS totals facilitates comparisons between MMP and NHSS data. In addition, collecting data from among all HIV diagnosed persons rather than among those out of care by a fixed definition allows flexibility in the choice of care definition and a richer description of care utilization, i.e., MMP describes not only persons who did not have two HIV care visits in the last year but also persons who had no visits at all during the 2-year medical record abstraction period. At the state and metropolitan area level, MMP estimates based on the population of persons diagnosed with HIV *residing* in the area as of a reference date are not directly comparable to estimates based on the population of persons *diagnosed* in that state. As with facility-based MMP data, state and metropolitan area estimates may be limited by sample size and require pooling of data across multiple years for adequate power.

In the first year of the pilot, 18% of the sample whose place of residence was established resided out of the jurisdiction of sampling and were excluded, because mechanisms for cross-jurisdictional data collection had yet to be established. Buskin, et al. have shown the importance of accounting for migration of persons living with HIV in other settings,[29] and it seemed likely that exclusion of these persons caused bias. This problem resulted from discrepancies between most recently recorded address in case surveillance and actual residence, and we took three steps to address it. First, we changed from using local case surveillance data to using national case surveillance data. This change alone was associated with a decrease from 18 to 14% in proportion of sampled persons with known residence who resided out of jurisdiction. Second, we established mechanisms to collect data despite cross-jurisdictional migration, which resulted in being able to recruit and collect data on persons who were not currently living in the jurisdiction in which they were sampled. Third, because response rates were lower for persons who moved out of the jurisdiction of sampling, we incorporated current residence into our non-response bias adjustment. These methods may prove useful to other surveillance systems or research efforts.

Our data show substantial variation in success of contacting sampled persons between subgroups. Not surprisingly, those who were out of care, had unsuppressed viral load, or who had moved out of the project area of sampling were more difficult to contact. Variables known to be associated with reporting delay in NHSS also predicted reporting delay for CSBS. Being diagnosed in a less populous area and having no history of AIDS were particularly strongly associated with increased reporting delay. These strong associations underscore the importance of weighting adjustments to reduce bias due to non-response and differential reporting delay. Nevertheless, our statistical adjustments depend on our ability to characterize differential response. We used NHSS data for this purpose, and in some cases variables were incomplete, e.g., race or transmission risk, and the data quality—particularly for laboratory data—varied by jurisdiction. Of particular importance was the adjustment for non-response based on HIV care status. We used NHSS laboratory data as a proxy for being out-of-care. Though viral load surveillance has been steadily improving nationwide, these methods have limited application in areas that do not have mature HIV laboratory reporting systems. Furthermore, we cannot be assured that adjustments based on these proxy data fully capture differences in non-response among people more or less engaged with their HIV care providers. Indeed, while laboratory results and medical record review are certainly correlated, the relationship is imperfect [32, 33]. In project areas with new or absent laboratory reporting systems, adjustments for care utilization would be indirect and reliant on adjustment for other factors that can be measured in the local NHSS data and may correlate with care utilization. Thus, it is possible that care utilization estimates in project areas without mature laboratory reporting systems would be biased due to incomplete adjustment. Finally, it should be mentioned that weighting not only makes point estimates more accurate through adjustment of bias, but it avoids Type 1 errors that may result when the investigator does not take into the account the systematic nature of non-response, i.e. investigators may inappropriately draw a conclusion of statistical significance if appropriate weighting and analysis methods are not used [34].

The necessity of excluding data from one state due to unforeseen circumstances further limited our ability to generalize the findings. Additionally, viral load reporting is one source of updated contact information for patients, and recruitment may be more challenging in states without mature viral load reporting systems. More generally, this manuscript is limited in its ability to generalize beyond the four included public health jurisdictions. CSBS sites were chosen through a competitive application process to which a minority of jurisdictions applied; it is possible that these applicants had more experience in use of HIV surveillance data for programmatic purposes, and extension of these methods to other jurisdictions may be more challenging. While Washington State data are used as an example, the most prominent findings from this dataset were replicated in the others. Being out of care significantly and strongly predicted lack of contact in all jurisdictions, and residing in a different jurisdiction consistently made it much less likely that an interview would be performed, though this effect was not statistically significant in LA County.

Over the past decade, the use of HIV surveillance data has shifted from a system in which information on individuals travelled from the HIV care provider to the surveillance system and was only used in aggregate to describe the population of persons diagnosed with HIV infection to one in which surveillance data are increasingly being used to reach out to persons with diagnosed HIV for the purpose of improving public health efforts in HIV. The methods employed by CSBS require the maintenance and use of personally identifiable data by the participating health department for recruitment. Although access and use of these data was strictly limited to staff of participating health departments for a specific public health use, the potential for breach in confidentiality exists and even using them to make contact could be considered by some to be an invasion of privacy; persons living with HIV do not have the opportunity to pre-emptively opt out of this initial contact. We further recognize that failure to use surveillance data to improve the health of people living with HIV and prevent transmission, now that effective methods exist, is ethically perilous.[35]To mitigate these challenges, we ensured that participation was fully voluntary, and we used rigorous security standards and a schedule for destruction of information when it was no longer necessary. No breaches of confidentiality occurred during the demonstration project.

When the national MMP surveillance system adapted the CSBS demonstration project’s methods, two modifications were implemented. First, considering the implications for statistical power of a more complex sampling design, sampling was not stratified by time since diagnosis as was done for the demonstration project. Second, weighting included an additional adjustment factor to account for the probability proportional to size selection of MMP states, similar to adjustments previously performed for MMP data[14] as this stage of sampling has not changed. The new MMP methods allow creation of national estimates for the entire population of adults living with a diagnosis of HIV in the United States, both receiving and not receiving HIV care. Interview and MRA data collected from persons not receiving HIV care, or marginally in care, will allow a deeper understanding of the reasons for failures in care engagement and better targeting of prevention resources. Insofar as good public health data drive good public health programs, our hope is that these methodological improvements bring us one step closer to controlling HIV in the United States.

## Supporting information

S1 Table Variables included in final models predicting successful contact by project area and year, Case-Surveillance-Based Sampling demonstration project 2012–2014. (DOCX)

S2 Table Variables included in final models predicting interview response among contacted persons by project area and year, Case-Surveillance-Based Sampling demonstration project 2012–2014. (DOCX)

S3 Table Variables included in final models predicting reporting delay project area and year, Case-Surveillance-Based Sampling demonstration project 2012–2014. (DOCX)
