# Supplementary material for: Methods to include persons living with HIV not receiving HIV care in the Medical Monitoring Project
Source: PLoS One. 2019 Aug 1;14(8):e0219996. doi: 10.1371/journal.pone.0219996 (PMC6675081; doi:10.1371/journal.pone.0219996)
Supplement: S1 Table — (DOCX) [file pone.0219996.s001.docx]

S1 Table: Variables included in final models predicting successful contact by project area and year, Case-Surveillance-Based Sampling demonstration project 2012-2014

|  | **2012** | | | | **2013** | | | | **2014** | | | |
| --- | --- | --- | --- | --- | --- | --- | --- | --- | --- | --- | --- | --- |
| Variable | LAC | NYC | SFO | WA | LAC | NYC | SFO | WA | LAC | NYC | SFO | WA |
| AIDS status |  |  |  |  | X | X | X | X | X | X | X | X |
| Care status |  |  |  | X | X | X | X | X | X | X | X | X |
| Number jurisdictions reporting | X | X | X | X | X | X | X | X | X | X | X | X |
| Race/Ethnicity: Hispanic | X | X | X | X |  |  |  |  | X | X | X | X |
| Race/Ethnicity: Non-Hispanic Black | X | X | X | X |  |  |  |  |  |  |  |  |
| Race/Ethnicity: Non-Hispanic White | X | X | X | X |  |  |  |  | X | X | X | X |
| Residence in project area of sampling^1^ | N/A | N/A | N/A | N/A | X | X | X | X | X | X | X | X |
| Sex | X | X | X | X |  |  |  |  | X | X | X | X |
| Suppressed HIV viral load^2^ | X | X | X | X | X | X | X | X | N/A | N/A | N/A | N/A |
| Time since last reported lab test^3^ | X | X | X |  | N/A | N/A | N/A | N/A | N/A | N/A | N/A | N/A |
| Transmission risk | X | X | X | X | X | X | X | X |  |  |  |  |
| Age at HIV diagnosis |  |  |  |  | X | X | X | X | X | X | X | X |
| Age on sampling date |  |  |  |  | X | X | X | X | X | X | X | X |
| Contact information age | X | X | X | X | X | X | X | X | X | X | X | X |
| Time since diagnosis | X | X | X | X | X | X | X | X |  |  |  |  |

^1^Indicates persons in 2013 and 2014 who actually resided in the project area in which they were sampled. In 2012, procedures had not yet been established to contact and interview out of jurisdiction persons so this variable was not applicable.

^2^This variable was excluded in 2014, because it was judged to be duplicative of care status, another measure of retention in care.

^3^This variable was excluded in 2013 and 2014, because it was judged to be duplicative of care status, another measure of retention in care.
