# Supplementary material for: Methods to include persons living with HIV not receiving HIV care in the Medical Monitoring Project
Source: PLoS One. 2019 Aug 1;14(8):e0219996. doi: 10.1371/journal.pone.0219996 (PMC6675081; doi:10.1371/journal.pone.0219996)
Supplement: S3 Table — (DOCX) [file pone.0219996.s003.docx]

S3 Table: Variables included in final models predicting reporting delay project area and year, Case-Surveillance-Based Sampling demonstration project 2012-2014

|  | **2012** | | | | **2013** | | | | **2014** | | | | |
| --- | --- | --- | --- | --- | --- | --- | --- | --- | --- | --- | --- | --- | --- |
| Variable | LAC | NYC | SFO | WA | LAC | NYC | SFO | WA | LAC | NYC | SFO | WA |  |
| AIDS History: AIDS Diagnosed After HIV | N/A | N/A | N/A | N/A | X | X | X | X | X | X | X | X |  |
| AIDS History: AIDS Diagnosed Concurrent with HIV | N/A | N/A | N/A | N/A | X | X | X | X | X | X | X | X |  |
| AIDS History Composite Variable | X | X | X | X | N/A | N/A | N/A | N/A | N/A | N/A | N/A | N/A |  |
| Facility of HIV Diagnosis, Type: Inpatient | N/A | N/A | N/A | N/A |  |  |  |  | X | X | X | X |  |
| Facility of HIV Diagnosis, Type: Missing | N/A | N/A | N/A | N/A | X | X | X | X | X | X | X | X |  |
| Facility of HIV Diagnosis, Type: Outpatient | N/A | N/A | N/A | N/A | X | X | X | X | X | X | X | X |  |
| Facility of HIV Diagnosis, Type: Composite Variable | X | X | X | X | N/A | N/A | N/A | N/A | N/A | N/A | N/A | N/A |  |
| Most Recent Jurisdiction of Residence Same as Jurisdiction Reporting Residence Data | X | X | X | X | X | X | X | X | N/A | N/A | N/A | N/A |  |
| Race/Ethnicity: Hispanic | N/A | N/A | N/A | N/A | X | X | X | X | X |  |  |  |  |
| Race/Ethnicity: Non-Hispanic Black | N/A | N/A | N/A | N/A |  |  |  |  | X | X | X | X |  |
| Race/Ethnicity: Non-Hispanic White | N/A | N/A | N/A | N/A | X | X | X | X | X | X |  |  |  |
| Race/Ethnicity: Other | N/A | N/A | N/A | N/A |  |  |  |  |  | X | X | X |  |
| Race/Ethnicity: Composite Variable | X | X | X | X | N/A | N/A | N/A | N/A | N/A | N/A | N/A | N/A |  |
| Sex | X | X | X | X | X | X | X | X | X | X | X | X |  |
| Transmission risk: MSM |  |  |  |  | X | X | X | X | X | X | X | X |  |
| Transmission risk: No Identified Risk |  |  |  |  | X | X | X | X | N/A | N/A | N/A | N/A |  |
| Transmission risk: Other Identified Risk |  |  |  |  | X | X | X | X | X | X | X | X |  |
| Urban Setting of HIV Diagnosis |  |  |  | X |  |  |  |  | X | X | X | X |  |
| Age at HIV diagnosis | X | X | X | X | X | X | X | X | X | X | X | X |  |
| HIV Diagnosis Date | X | X | X | X | X | X | X | X | X | X | X | X |  |
